# Development of Polyclonal Anti‐HBsAg IgY Antibodies and Comparative Evaluation of Glutaraldehyde Versus Periodate HRP Bioconjugation for Hepatitis B Diagnosis

**DOI:** 10.1155/bmri/6006257

**Published:** 2026-11-15

**Authors:** Felicienne Tchoufang Tchatchoua, Herman Philipe Nfombouot-Njitoyap, Yacouba Foupouapouognigni, Floriane Tekoutsap Manetsop, Arnaud Fondjo Kouam, Frédéric Nico Njayou, Simon Ngamli Fewou, Paul Fewou Moundipa

**Affiliations:** ^1^ Faculty of Health Science Université des Montagnes Bangangté Cameroon udesmontagnes.org; ^2^ Laboratory of Pharmacology and Toxicology Department of Biochemistry Faculty of Science University of Yaoundé 1 Yaoundé Cameroon uy1.uninet.cm; ^3^ Department of Biomedical Sciences Faculty of Health Sciences University of Buea Buea Cameroon ubuea.cm

**Keywords:** glutaraldehyde crosslinking, HBsAg detection, hepatitis B diagnosis, HRP bioconjugation, IgY antibodies, periodate oxidation

## Abstract

Hepatitis B virus remains a major global health burden, particularly in sub‐Saharan Africa, where affordable, specific and locally producible diagnostic tools are urgently needed. Conventional mammalian antibody‐based assays can suffer from high background signals and interference in human sera. We developed a polyclonal chicken IgY reagent against the immunodominant “a” determinant of HBsAg, using consensus peptides to limit immune escape: two 24‐amino‐acid consensus peptides from the major hydrophilic region were synthesised and used to immunise laying hens. Antigen‐specific IgY was extracted from egg yolks by salting‐out precipitation, purified by peptide‐affinity chromatography and conjugated to horseradish peroxidase by glutaraldehyde cross‐linking and by sodium periodate oxidation. SDS‐PAGE under reducing and non‐reducing conditions confirmed the structural integrity of the purified IgY. Both chemistries yielded functional conjugates, but the periodate‐oxidised conjugate gave a significantly higher signal across dilutions (Wilcoxon test, *p* = 0.016). In an adapted commercial sandwich ELISA applied to 10 HBsAg‐positive and 10 HBsAg‐negative sera, the in‐house IgY–HRP conjugates produced optical density profiles that differed from the commercial biotinylated system (repeated‐measures ANOVA, *p* < 0.0001) while separating positive from negative sera completely. Receiver‐operating‐characteristic analysis gave an area under the curve of 1.000 for all three detection systems, and at the Youden‐derived cut‐offs (OD450 0.77 for the glutaraldehyde and 0.80 for the periodate conjugate) both in‐house reagents reached 100% sensitivity and 100% specificity (95% CI 72.2%–100% for each, *n* = 10 per group). This work establishes a low‐cost, locally producible IgY‐based platform for HBsAg detection, offering a scalable alternative for HBV screening in resource‐limited, high‐prevalence settings.

## 1. Introduction

Hepatitis B virus infection continues to impose a staggering global health burden, with an estimated 254 million people living with chronic infection in 2022 and 1.2 million new infections occurring annually, resulting in 1.3 million hepatitis‐related deaths, 83% of which are attributable to HBV. Sub‐Saharan Africa bears a disproportionately heavy burden of hepatitis B, with approximately 65 million chronically infected individuals in the WHO African Region. This represents 63% of global new infections, and many countries, including Cameroon, report HBsAg seroprevalence rates consistently above 5%–8%. In these resource‐limited environments, late diagnosis, limited access to affordable testing and high rates of perinatal and horizontal transmission perpetuate cycles of chronic liver disease, cirrhosis and hepatocellular carcinoma. Effective control hinges on scalable, sensitive and specific diagnosis of hepatitis B surface antigen (HBsAg), which is the cornerstone serological marker of active infection. Yet conventional mammalian antibody‐based assays are often in short supply in high‐prevalence Sub‐Saharan African populations [1, 2].

Current commercial ELISA platforms predominantly use mammalian (IgG) polyclonal or monoclonal antibodies targeting the immunodominant “a” determinant within the major hydrophilic region (MHR; residues 99–169) of HBsAg. Although this epitope is highly conserved and immunogenic, it is also a hotspot for immune‐escape mutations driven by vaccination pressure, natural immunity or antiviral therapy. Such variants can compromise assay sensitivity and contribute to diagnostic failures.

Moreover, mammalian IgG reagents are prone to high background signals and interference in human sera because of cross‐reactivity with rheumatoid factors, complement components and Fc receptors, reducing specificity in endemic settings where comorbidities and polyclonal B‐cell activation are common [3].

Avian immunoglobulin Y (IgY), harvested non‐invasively from the yolks of immunised laying hens, offers a compelling alternative. Unlike mammalian IgG, IgY exhibits negligible cross‐reactivity with human complement, rheumatoid factor or Fc receptors, resulting in lower non‐specific binding and superior signal‐to‐noise ratios in human matrices [4]. IgY production is ethically advantageous and cost‐effective. Highly scalable hens yield gramme quantities of antibody annually with minimal animal distress, and the resulting antibodies display enhanced thermal and proteolytic stability. Prior studies have successfully applied anti‐HBsAg IgY in ELISA formats, demonstrating high sensitivity for HBsAg detection without the interference typically observed in mammalian systems [5]. Complementary work has extended IgY′s utility to related hepatotropic viruses, underscoring its versatility in resource‐constrained diagnostic pipelines [6]. IgY‐based immunoassays occupy one end of a broad and rapidly moving field of biosensing chemistry. At the other end, nucleic‐acid‐based platforms that couple CRISPR‐Cas effectors to signal‐amplification scaffolds are achieving very low limits of detection for viral and oncological targets [7–9]. Those platforms target nucleic acids rather than a circulating protein antigen and currently require instrumentation and cold‐chain reagents that are difficult to sustain in decentralised settings; the antibody‐based route pursued here is complementary to them, trading analytical sensitivity for reagent stability, low unit cost and local manufacturability.

To further mitigate escape‐variant risks, consensus peptide design derived from comprehensive mutational analyses of the MHR “a” determinant has emerged as a strategy to elicit broader polyclonal responses that better accommodate sequence diversity. Earlier work by our group established the foundational immunogenicity of these consensus peptides, showing superior responses with peptide mixtures compared with single sequences [10]. However, critical gaps persist: (1) limited integration of consensus‐peptide IgY into full diagnostic workflows using clinical sera from high‐burden SSA cohorts; (2) incomplete optimisation of downstream bioconjugation steps required for sensitive enzyme‐linked detection; and (3) direct head‐to‐head comparisons of conjugation chemistries to maximise performance.

Horseradish peroxidase (HRP) conjugation remains a rate‐limiting step for IgY‐based ELISA reagents. Two established chemistries predominate: homobifunctional glutaraldehyde cross‐linking (targeting amine groups) and heterobifunctional sodium periodate (NaIO₄) oxidation (generating aldehydes on HRP carbohydrate moieties, which undergo reductive amination with antibody lysines). Literature consistently indicates that periodate‐mediated coupling provides more attachment sites, higher labelling efficiency and better preservation of both paratope integrity and enzyme activity. This often translates to superior assay sensitivity compared with glutaraldehyde, which can induce over‐crosslinking and denaturation at higher concentrations [11, 12]. Yet these comparisons have rarely been performed for avian IgY or consensus‐peptide anti‐HBsAg systems, leaving open the question of which chemistry best suits locally produced reagents for SSA diagnostic needs [12, 13]. This study addresses these gaps by developing a complete, end‐to‐end polyclonal anti‐HBsAg IgY platform tailored for HBV diagnosis in high‐prevalence settings.

## 2. Methodology

### 2.1. Antigen Synthesis and Sample Acquisition

Consensus peptide sequences for HBsAg were derived from prior mutational analyses of [10], focusing on the MHR (positions 99–169) containing the immunodominant “a” determinant. This region was selected for its high immunogenicity, making it ideal for both vaccine and diagnostic development, whereas the consensus design mitigates escape mutations induced by immune pressure or vaccination [10]. Two 24‐amino‐acid peptides (Peptide 1: 2454.75 Da, 95.3% purity; Peptide 2: 2530.03 Da, 96.2% purity) were synthesised by GenScript (Piscataway, New Jersey, United States) and provided as lyophilised white powder. Their sequences were as follows: Peptide 1: CTTPAQGNSMYPSCCCSKPSDGNC, and Peptide 2: CMICVQGTSMFPSCCCTKPTDGNC. Human sera, positive and negative for HBsAg, were obtained from the Blood Bank of the Yaoundé Military Hospital. A total of 20 anonymised residual serum specimens were included, comprising 10 HBsAg‐positive and 10 HBsAg‐negative samples (designated PS 1–PS 10 and NS 1–NS 10 in Figure 8). Donors had a median age of 28 years (range [25–32]) and a male‐to‐female ratio of [1:1]. Samples were aliquoted and stored at −20°C until use, and freeze‐thaw cycles were limited to [2]. The sera were de‐identified blood‐donor samples released to the study without accompanying demographic or clinical information, HBV viral load and genotype and time since infection could not be documented. HBsAg test was performed using Fortress Diagnostics ELISA kits (Antrim, United Kingdom), detecting HBsAg via HRP‐conjugated anti‐HBsAg IgG and 3, 3^′^, 5, 5^′^‐tetramethylbenzidine (TMB) substrate; optical density (OD) ≥ 1 at 450 nm indicated positivity. This commercial assay served as the reference classification against which the diagnostic performance of the in‐house conjugates was assessed (Section 2.10).

### 2.2. Hen Immunisation

Three 6‐month‐old Lohmann Brown‐Classic laying hens were sourced from the AGROCAM poultry complex in Foumbot, Cameroon. The hens were housed in cages measuring 150 × 100 × 80 cm within the animal facility of the Laboratory of Pharmacology and Toxicology, Faculty of Sciences, University of Yaoundé I. They received layer feed (250 ± 10 g per hen per day) and had unlimited access to tap water. To enhance immunoreactivity, a mixture of the two peptides was used, as prior studies had shown superior responses compared with individual peptides [10]. The first group of three hens was immunised intramuscularly in the wing with 1 mg/mL peptide mixture emulsified in complete Freund′s adjuvant (Catalogue No. F5881) for the primary dose, followed by incomplete Freund′s adjuvant (Catalogue No. F5506) for boosters at 2, 4 and 6 weeks after the first immunisation. The second group of three hens, used as controls, was immunised with complete Freund′s adjuvant without any antigen for the primary dose, followed by incomplete Freund′s adjuvant only without antigen for the boosters. Complete and incomplete Freund′s adjuvant were purchased from Sigma–Aldrich (St. Louis, Missouri, United States). Eggs were collected between Weeks 3 and 5 after the booster, pooled (two eggs per pool, three pools per week) and stored at 4°C. This timeline was chosen based on peak IgY deposition in yolks, typically 3–6 weeks postimmunisation.

### 2.3. IgY Extraction From Egg Yolks

IgY was isolated as previously described by [10] [10]. Briefly, egg yolks were carefully separated from the whites, rinsed with distilled water and gently squeezed to remove residual albumen. The yolks were then diluted 1:1 (v/v) with 1X phosphate buffered saline (PBS) containing 0.1% sodium azide. One volume of this yolk‐PBS mixture was further diluted with eight volumes of distilled water, the pH was adjusted to 5.0 with 0.5 N HCl, and the solution was frozen at −20°C. After thawing, the mixture was centrifuged (4000 × *g*, 30 min at 4°C), and the supernatant was filtered through a 0.22 *μ*m pore filter. The filtrate was then supplemented with 8.8% (w/v) sodium chloride (NaCl), and the pH was adjusted to 4.0 using 0.5 N hydrochloric acid (HCl). The solution was stirred for 2 h at room temperature (RT) and centrifuged (3700 × *g*, 30 min, 4°C). The resulting pellet was resuspended in PBS 1X + 0.1% sodium azide at 10% of the original filtrate volume to obtain the soluble fraction enriched in antigen‐specific IgY.

### 2.4. Protein Quantification

Total protein was quantified using the Bradford assay [14] and bovine serum albumin (BSA) standards (0–300 *μ*g/mL).

### 2.5. Electrophoretic Analysis

The purity and identity of the isolated IgY were verified by SDS‐PAGE, which separates proteins based on their molecular weight through a polyacrylamide gel matrix under an electric field. Briefly, gels were prepared by first casting a 10% resolving gel between glass plates. The surface was overlaid with ethanol to promote even polymerisation, which was allowed to occur at RT for 20 min. After removing the ethanol, a 5% stacking gel (2 mL) was poured, and a 1 mm comb was inserted to form sample wells.

Antigen‐specific IgY samples were analysed under two conditions. For reducing SDS‐PAGE, samples were mixed with 5x Laemmli buffer containing 1% (v/v) DTT and heated at 100°C for 10 min; for non‐reducing SDS‐PAGE, the identical Laemmli buffer was used without any reducing agent, and the samples were not heated. All samples were briefly centrifuged (5000 rpm, 2 min) before loading. Then, the volume corresponding to 50 *μ*g of protein and prestained molecular weight markers (PageRuler Prestained Protein Ladder, 10‐180 kDa; Catalogue No. 26617, Thermo Fisher Scientific) was loaded into the wells.

Migration occurred at 100 V (stacking gel, 10–15 min) and 150 V (resolving gel, 50–60 min). Gels were stained with Coomassie brilliant blue R‐250 (0.1% in water:ethanol:acetic acid, 45:45:10) for 30 min and destained in water:acetic acid:ethanol (80:10:10) overnight. Under non‐reducing conditions, this method confirms the integrity of the intact ~180 kDa IgY molecule, while under reducing conditions it resolves into its constituent heavy and light chains; both conditions detect copurifying impurities, thereby guiding the purification strategy.

### 2.6. Immunoreactivity Assessment by Indirect ELISA

The immunoreactivity of the extracted IgY against its target antigens was assessed using an indirect ELISA. In brief, 96‐well microtiter plates were coated overnight at 4°C with 100 *μ*L of 50 mM carbonate‐bicarbonate buffer (pH 9.6) containing 2 *μ*g of the antigen (combination of both peptides). The plates were then blocked with 100 *μ*L of 5% (w/v) skimmed milk in PBS 1x, pH 7.4, for 1 h at RT. After blocking, 100 *μ*L of serially diluted specific IgY (starting at 10 *μ*g) was added to each well and incubated for 2 h. The plates were washed twice with PBS containing 0.05% Tween‐20 (PBST). Subsequently, 100 *μ*L of HRP‐conjugated goat antichicken IgG secondary antibody (Cat A16054) was added and incubated for 1 h, followed by two additional washes with PBST. The colourimetric reaction was developed by adding 100 *μ*L of TMB substrate and incubating it for 15 min in the dark. The reaction was stopped by adding 50 *μ*L of 2 M sulphuric acid (H2SO₄), and the OD was measured at 450 nm. The endpoint titre was defined as the highest dilution (or lowest concentration) of specific IgY that produced an OD value greater than twice the OD obtained with the highest concentration of IgY from nonimmunised hens.

### 2.7. Purification of IgY by Affinity Chromatography

To couple the antigen to the cyanogen bromide (CNBr) activated sepharose gel for IgY affinity purification, 10 *μ*g of antigen was first applied in a dialysis tube and dialysed in PBS at 4°C for 2 h. The process was repeated twice. The dialysis tube was then placed in the coupling buffer (0.1 M sodium bicarbonate NaHCO3, pH 8.3, containing 0.5 M NaCl) and dialysed at 4°C for 2 h. The dialyzed antigen was transferred into 50 *μ*L of CNBr‐activated sepharose gel and agitated at 4°C overnight. The mixture was centrifuged at 3000 *g* for 5 min, and the supernatant was discarded. The gel was washed with 1 mL of coupling buffer, centrifuged at 3000 *g* for 5 min and the supernatant was discarded. This washing step was repeated five times. Blocking was performed by adding 1 mL of blocking buffer (0.1 M Tris‐HCl, pH 8.0) and incubating at RT for 2 h. The mixture was centrifuged for 5 min at 3000 *g*, and the supernatant was discarded. The gel was washed with 300 *μ*L of 0.1 M sodium acetate (CH_3_COONa) buffer, pH 4.0, containing 0.5 M NaCl, centrifuged at 3000 *g* for 5 min and the supernatant was discarded. It was then washed with 300 *μ*L of 0.1 M Tris‐HCl buffer, pH 8.0, containing 0.5 M NaCl and centrifuged at 3000 *g* for 5 min. The supernatant was discarded, and the washing steps (sodium acetate and Tris‐HCl buffers) were repeated three times. Finally, the gel was washed with 300 *μ*L of 1X PBS, centrifuged at 3000 *g* for 5 min and the supernatant was discarded.

Following the preparation of the antigen‐coupled CNBr‐activated sepharose gel, 2.5 mL of protein extracts were filtered through a 0.45 *μ*m membrane. The filtered extracts were then added to the sepharose and gently agitated for 1 h at 4°C to allow binding of the IgY. The resulting mixture was transferred into a filtration column and centrifuged at 3000 *g* for 5 min. The obtained filtrate was discarded, after which the gel was washed with 300 *μ*L of 1X PBS and centrifuged at 3000 *g* for 3 min; this PBS washing step was repeated five times. The filtration column was subsequently transferred into a new tube containing 37.5 *μ*L of 1 M Tris. Next, 150 *μ*L of elution buffer (0.17 M glycine‐HCl, pH 2.5) was added directly to the column and centrifuged at 3000 *g* for 5 min to elute the bound antibodies. The purified antibodies were then dialysed in 1X PBS at 4°C for 2 h, and the process was repeated three times. Finally, the purified IgY antibodies were stored at −20°C.

### 2.8. Bioconjugation of Purified IgY to HRP

All the bioconjugation was done according to the modified protocols of [15].

#### 2.8.1. Bioconjugation Using Homoconjugation

The purified IgY was conjugated to HRP using glutaraldehyde, a bifunctional crosslinker selected for its ability to form stable covalent bonds via Schiff base formation while minimising enzyme denaturation at low concentrations, as illustrated in Figure 1. The conjugation solution (prepared at final concentrations of 100 *μ*g/mL IgY plus 250 *μ*g/mL HRP in 0.02 M phosphate buffer containing 0.15 M NaCl at pH 7.4) was mixed with 4 *μ*L of either a 25% or a 12.5% (w/v) aqueous glutaraldehyde stock solution per 400 *μ*L of conjugation mixture. The percentages quoted throughout refer to the concentration of the stock solution, not to the reaction mixture: the corresponding final concentrations in the reaction were 0.248% (w/v) (24.7 mM) and 0.124% (w/v) (12.4 mM), respectively. These mixtures were then incubated for 2 h at 4°C before the reaction was stopped by the addition of 0.2 M glycine in 0.5 M carbonate buffer at pH 9.4 and then further incubated for 2 h at RT. The resulting conjugates were stored at + 4°C until required analysis. Their reactivity was subsequently evaluated by direct ELISA as previously described, in which microtiter plates were first coated with 1 *μ*g/mL of the peptides, blocked and then incubated with serial dilutions of each conjugate for 1 h at 37°C. The plates were developed with TMB substrate for 30 min, the enzymatic reaction was stopped with H2SO₄, and the OD was read at 450 nm. Taking relative molecular weights (Mr) = 180 kDa for IgY and 44 kDa for HRP, the input molar ratio of HRP to IgY in the glutaraldehyde reaction was 10.2:1. The conjugate prepared with the 12.5% glutaraldehyde stock (0.124% final) yielded optimal enzymatic activity while effectively reducing non‐specific crosslinking.

**Figure 1:**
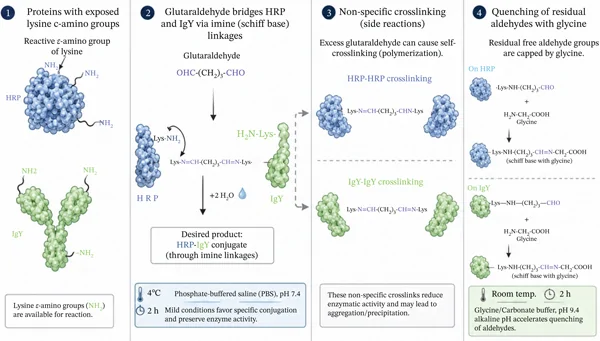
Chemical representation of homoconjugation with glutaraldehyde.

#### 2.8.2. Bioconjugation by Heteroconjugation

The chemistry of reactions under this bioconjugation is illustrated in Figure 2. The enzyme was first dissolved at 20 mg/mL in buffer containing 0.01 M sodium phosphate, 0.15 M NaCl, pH 7.2. A freshly prepared 0.088 M NaIO₄ solution (protected from light) was added at 100 *μ*l per mL of enzyme solution to achieve an 8 mM final periodate concentration. The mixture reacted in the dark for 15–20 min at RT, during which HRP changed colour from brownish gold to green. The reaction was immediately stopped by adding 0.1 mL of glycerol per mL of mixture. The oxidised enzyme was then adjusted to 10 mg/mL in the same phosphate buffer. The periodate‐activated enzyme was stored frozen for long‐term stability and not kept in solution at RT or + 4°C because of the risk of self‐polymerisation.

**Figure 2:**
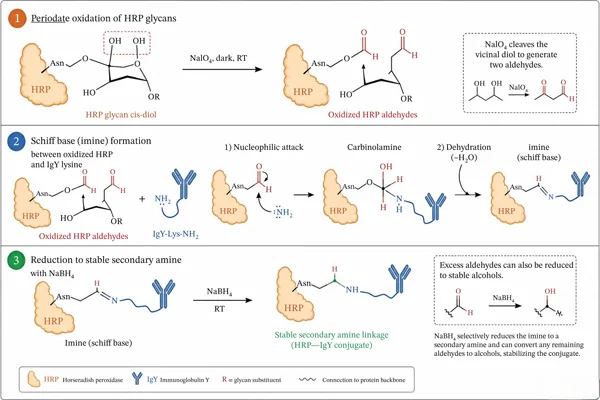
Chemical representation of heteroconjugation based on sodium periodate.

Conjugation was performed by reductive amination between the periodate‐oxidised HRP and the antibody (IgY). The IgY was dissolved at 10 mg/mL in 0.2 M sodium bicarbonate, pH 9.6, for high enzyme‐to‐antibody ratios and maximal incorporation. Equal volumes of the antibody and oxidised enzyme solutions were mixed, resulting in a 3.75:1 molar excess of HRP over IgY, calculated for the same molecular masses of 165 kDa for IgY and 44 kDa for HRP that were used for the glutaraldehyde reaction (9.4:1). The molar labelling degree of the finished conjugates, that is the number of HRP molecules incorporated per IgY molecule, was not determined spectrophotometrically; the two chemistries were therefore compared functionally, by direct ELISA titration of equal conjugate dilutions on the same antigen‐coated plate. The mixture was left to react for 2 h at RT. Excess periodate was stopped by adding 10 *μ*L of 5 M glycine per mL of reaction solution, followed by a 30‐min incubation. Remaining aldehyde groups were then blocked with 50 *μ*L of 1 M ethanolamine (pH 9.6) per mL of reactive medium for another 30 min at RT. The final conjugate was stored at 4°C and evaluated by direct ELISA. For this, 1 *μ*g/mL of the mixture of the two peptides was fixed to the bottom of a microtitration plate for overnight incubation. The plate was washed four times with 200 *μ*L of washing buffer, then 100 *μ*L of blocking buffer was added to the wells. After 1 h of incubation at 37°C, the plate was washed as previously described, and 100 *μ*L of the bioconjugation product (diluted in a series with a ratio of 2) was added to the wells and incubated for 1 h at 37°C. After washing to eliminate any unfixed products, the antigen‐IgY–HRP complex was revealed by adding 100 *μ*L of TMB. After 30 min of incubation, 100 *μ*L of H2SO₄ was added to stop the reaction, and the OD was read at 450 nm.

### 2.9. Evaluation of the Diagnostic Capacity of Conjugated IgY

To evaluate the diagnostic performance and binding capacity of the conjugated IgY antibodies, a commercial sandwich ELISA kit from Elabscience (Houston, Texas, United States) was adapted by incorporating the in‐house conjugated IgY detection antibodies. A 96‐well micro‐ELISA plate was divided into three distinct sections, each dedicated to one of the following detection antibodies: (1) the biotinylated detection antibody supplied by the manufacturer (used as the reference standard), (2) the IgY–HRP conjugate prepared via glutaraldehyde cross‐linking and (3) the IgY–HRP conjugate prepared via the NaIO₄ oxidation method.

The assay was performed as follows. First, 100 *μ*L of blank or appropriately diluted positive and negative samples (at a ratio of ½) were added in duplicates to the corresponding wells of the precoated micro‐ELISA plate with capture antibodies. The plate was then sealed with an adhesive sealer and incubated for 90 min at 37°C to allow antigen capture. The liquid was then decanted, and the plate was washed five times with 350 *μ*L of wash buffer per well to remove unbound material.

Next, prepared at the optimised working concentration, 100 *μ*L of each detection antibody solution was added to the respective sections of the plate: the manufacturer′s biotinylated detection antibody to the first section, the glutaraldehyde‐conjugated IgY–HRP to the second section and the periodate‐conjugated IgY–HRP to the third section. The plate was sealed with a fresh adhesive sealer and incubated for 1 h at 37°C. Following incubation, the plate was washed four times with 350 *μ*L of wash buffer per well.

For the wells containing the biotinylated detection antibody, 100 *μ*L of the streptavidin‐HRP conjugate working solution (provided in the kit) was added. No other conjugate was required for the IgY–HRP sections, as the HRP was already covalently linked to the IgY. The plate was sealed again and incubated for 30 min at 37°C. After this step, the plate was washed five times with 350 *μ*L of wash buffer per well.

Subsequently, 100 *μ*L of TMB substrate reagent was added to all wells. The plate was sealed, protected from light and incubated for approximately 15 min at 37°C to allow colour development. The enzymatic reaction was then terminated by adding 50 *μ*L of stop solution (H2SO₄ 2 M) to each well, done in the same order as the substrates to ensure consistent reaction times. Immediately afterwards, the OD of each well was measured at 450 nm using a preheated microplate reader.

### 2.10. Determination of the Assay Cut‐Off and Statistical Analysis

All OD₄50 values are reported as mean ± standard deviation (SD). Each experiment was performed on two independent assay runs. Within each run, the indirect ELISA of crude IgY and of the chromatography fractions was read in three replicate wells, the direct ELISA of IgY–HRP conjugates and all conjugate titrations in two replicate wells, and the adapted sandwich ELISA (including each clinical serum) in duplicate wells. The number of replicates and whether they represent technical replicates or independent runs are stated in each figure legend.

The diagnostic cut‐off of the adapted sandwich ELISA was determined separately for each of the three detection systems from the blank‐corrected OD₄50 values of the 10 HBsAg‐positive and 10 HBsAg‐negative sera. For each detection antibody, a receiver‐operating‐characteristic (ROC) curve was constructed, and the area under the curve (AUC) was calculated. The optimal threshold was identified by maximising the Youden index (J = sensitivity + specificity–1). Because the positive and negative distributions were completely separated, the cut‐off was fixed at the midpoint of the separating interval; the conventional serological threshold (mean OD₄50 of the negative sera + 3 SD) is reported alongside it as a more conservative alternative. Sensitivity, specificity, predictive values and overall accuracy were calculated against the Fortress Diagnostics reference classification, with 95% confidence intervals (CIs) obtained by the Wilson score method. Signal‐to‐cut‐off (S/CO) ratios are reported for the weakest positive and the strongest negative serum in the panel.

Paired titration curves were compared across dilutions with the Wilcoxon matched‐pairs signed‐rank test. The three detection systems of the adapted sandwich ELISA were compared by one‐way repeated‐measures ANOVA, separately for the positive and the negative sera, followed by Bonferroni‐corrected pairwise tests (paired *t* tests or Wilcoxon signed‐rank tests as appropriate). Agreement between each in‐house conjugate and the commercial biotin/streptavidin system was assessed by Pearson correlation and Bland–Altman analysis. Comparisons of total protein between chromatography fractions used the Mann–Whitney *U* test. All tests were two‐sided, and *p* < 0.05 was considered significant. Analyses were performed with GraphPad Prism 10 (GraphPad Software, Boston, Massachusetts, United States).

## 3. Results and Interpretation

The initial characterisation of the extracted IgY antibodies was performed by SDS‐PAGE under both non‐reducing and reducing conditions (Figures 3 and 5B). SDS‐PAGE is denaturing under both conditions; the two preparations differ only in the presence or absence of a reducing agent. Under non‐reducing conditions, a clear band corresponding to an MW of approximately 180 kDa was observed, which represents the intact tetrameric structure of the IgY molecule. When the same samples were analysed under reducing conditions, two distinct bands appeared: one at 72 kDa corresponding to the heavy chains and another at 26 kDa corresponding to the light chains. These MWs are typical for avian IgY antibodies, which are structurally similar yet slightly larger than mammalian IgG. This result confirms that the extraction process successfully yielded properly assembled IgY molecules with intact interchain disulphide bonding under non‐reducing conditions, whereas the addition of a reducing agent effectively dissociated them into their constituent polypeptide chains. These electrophoretic profiles demonstrate both the purity and structural integrity of the isolated antibodies, indicating that the IgY maintained its functional architecture throughout the initial extraction steps.

**Figure 3:**
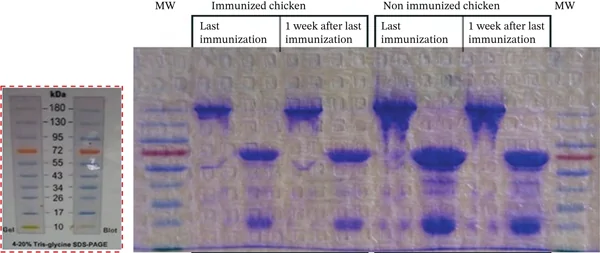
Representation of IgY extracts on a polyacrylamide gel. Dashed represents the manufacturer′s reference image of the PageRuler Prestained Protein Ladder (Thermo Scientific, Cat. No. 26616), run on a 4%–20% Tris‐glycine SDS‐PAGE gel. This image is shown only to help assign molecular weights. The molecular‐weight (MW) lanes in the gels contain the same ladder loaded onto each gel. Reducing SDS‐PAGE of crude egg‐yolk IgY extracts. Lanes 1 and 10 contain prestained molecular‐weight markers (PageRuler, 10–180 kDa). Lanes 2–5 show samples from immunised hens: Lanes 2 and 3 contain eggs collected at the time of the last immunisation, and Lanes 4 and 5 contain eggs collected 1 week after the last immunisation. Lanes 6–9 show samples from nonimmunised control hens: Lanes 6 and 7 contain eggs collected at the time of the last immunisation, and Lanes 8 and 9 contain eggs collected 1 week after the last immunisation. The lane assignments in this legend correspond to the annotations on the gel image. The gel shown is a representative example from two independent extractions. Each lane was loaded with 50 *μ*g of total protein, and the gel was stained with Coomassie brilliant blue R‐250.

Following structural characterisation, the immunoreactivity of the crude IgY preparation was evaluated using an indirect ELISA (Figure 4). In this assay, the target antigen was coated onto the plate at a concentration of 1 *μ*g/mL, and varying concentrations of IgY were tested for binding. The results showed a clear dose‐dependent increase in OD, with higher IgY concentrations producing stronger signals. This pattern indicates that the extracted antibodies specifically recognise and bind to the immobilised antigen in a concentration‐dependent manner. The successful immunoreactivity of the unpurified IgY demonstrates that the immunisation of the hens effectively elicited a specific polyclonal antibody response. These findings establish that the crude extract already contained functional antigen‐specific antibodies, providing a solid foundation for subsequent purification and labelling steps. At and above 6.25 *μ*g/mL, the signal from immunised hens exceeded that of the nonimmunised controls by 1.9‐fold to 5.5‐fold, and the endpoint titre defined in Section 2.6 corresponded to 25 *μ*g/mL of crude IgY (Wilcoxon matched‐pairs signed‐rank test across the dilution series, *p* = 0.0122).

**Figure 4:**
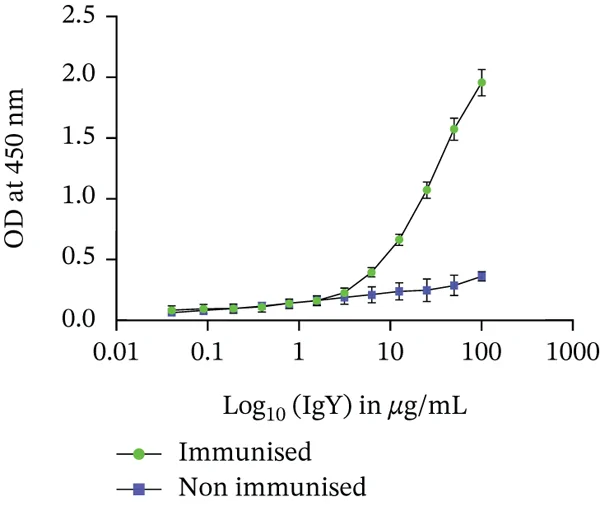
Immunoreactivity of serially diluted crude IgY from immunised and nonimmunised hens by indirect ELISA. Plates were coated with 1 *μ*g/mL of the two‐peptide mixture. Symbols are the mean, and error bars represent the standard deviation (SD) (three technical replicates in each of two independent assay runs). The dotted line marks the endpoint criterion (twice the OD450 obtained with the highest concentration of IgY from nonimmunised hens); the endpoint titre corresponded to Log_10_ 25 *μ*g/mL (1.39) of crude IgY. Signals from immunised and nonimmunised hens diverged from 6.25 *μ*g/mL upward (Wilcoxon matched‐pairs signed‐rank test across the 12 concentrations, *p* = 0.0122).

The purification of IgY was achieved through affinity chromatography using CNBr‐activated sepharose resin with immobilised peptides. The eluted fractions exhibited high purity with a clear 180 kDa band and minimal contaminants on electrophoresis. Although the flow‐through filtrates contained higher total protein concentrations, they showed significantly lower antigen‐specific immunoreactivity. Protein quantification using the Bradford assay (Figure 5A) surprisingly indicated higher total protein concentrations in the filtrate fractions compared with the eluates. Analysis of the fractions by non‐reducing and reducing SDS‐PAGE (Figure 5B) revealed that the eluted fractions contained highly pure IgY with virtually no visible impurities, whereas the flow‐through filtrates showed the presence of several contaminating proteins. However, when immunoreactivity was assessed by indirect ELISA (Figure 5C), the first eluate (Eluate 1) exhibited the strongest specific signal against the target antigen, confirming effective enrichment of functional, antigen‐specific IgY. This contrast highlights that although the filtrate contained more total protein, most of it consisted of non‐specific contaminants. In contrast, the affinity‐purified eluate was highly enriched in antigen‐specific IgY. These results confirm the effectiveness of the affinity chromatography step in isolating high‐purity, functional antibodies, which is essential for preventing unwanted side reactions during subsequent bioconjugation.

**Figure 5:**
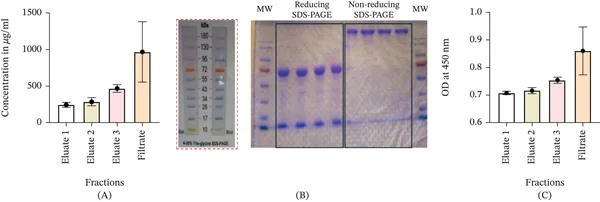
Evaluation of IgY purification by affinity chromatography. (A) Total protein in each fraction by the Bradford assay. (B) Non‐reducing and reducing SDS‐PAGE of the purified IgY. The dashed panel on the left is the manufacturer′s reference image of the ladder (Thermo Scientific, Cat. No. 26616). It is shown for size assignment only and is not part of the experimental gel. Under reducing conditions, the preparation resolves into two main bands, at approximately 72 kDa (heavy chain) and 26 kDa (light chain). Under non‐reducing conditions, the intact immunoglobulin migrates as a single band at approximately 180 kDa. MW, PageRuler Prestained Protein Ladder, loaded on the left and right of the gel. (C) Antigen‐specific immunoreactivity of each fraction by indirect ELISA. In panels (A) and (C), bars are the mean, and error bars are the SD of *n* = 6 replicates (three technical replicates in each of two independent purifications).

The purified IgY was then successfully conjugated to HRP using glutaraldehyde, applied at two stock concentrations (25% and 12.5%, corresponding to 0.248% and 0.124% in the reaction mixture), and, separately, using NaIO₄ oxidation. The performance of the resulting IgY–HRP conjugates was evaluated by direct ELISA through serial dilutions (Figure 6). The assay demonstrated strong immunoreactivity, particularly at low dilutions (high conjugate concentrations), indicating that both the antigen‐binding sites of the IgY and the enzymatic activity of the HRP were well preserved after conjugation.

**Figure 6:**
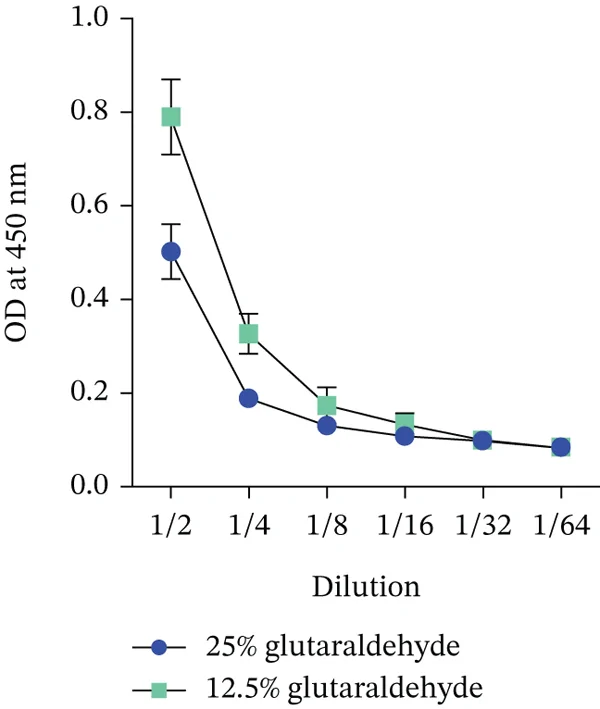
Direct ELISA immunoreactivity of IgY–HRP conjugates prepared with 25% and 12.5% glutaraldehyde stock solutions (0.248% and 0.124% final concentrations in the reaction). Symbols are the mean, and error bars are the SD of *n* = 4 replicates (two replicate wells in each of two independent conjugations). Wilcoxon matched‐pairs signed‐rank test across the six dilutions, *p* = 0.0312.

The results indicate that both conjugation conditions were successful, as both conjugates retain antigen‐binding activity and enzymatic function. However, the 12.5% glutaraldehyde concentration is clearly more efficient for labelling IgY with HRP.

The higher OD values obtained with the 12.5% conjugate suggest better preservation of the antibody′s antigen‐binding sites (paratopes), higher labelling efficiency (more HRP molecules successfully attached per IgY without causing aggregation or inactivation) and reduced risk of over‐crosslinking or denaturation of the antibody or enzyme, which can occur when using a higher concentration of glutaraldehyde (25%). This is consistent with the statistical analysis: across the six dilutions of the titration curve, the conjugate prepared with the 12.5% glutaraldehyde stock gave significantly higher signals than the one prepared with the 25% stock (Wilcoxon matched‐pairs signed‐rank test, *p* = 0.0312), the advantage being largest at the lowest dilutions (1.6‐fold to 1.7‐fold at 1/2 and 1/4).

The periodate method is clearly superior in terms of sensitivity and overall immunoreactivity, as shown in Figure 7. The periodate conjugate gave a 1.4‐fold higher OD450 than the glutaraldehyde conjugate at the neat dilution, and its advantage widened with dilution to 2.8‐fold at 1/64, a mean of 2.2‐fold across the seven dilutions, so that usable signals were maintained over a wider dilution range. This suggests better labelling efficiency, higher HRP incorporation per antibody or better preservation of antibody binding activity with the periodate oxidation approach.

**Figure 7:**
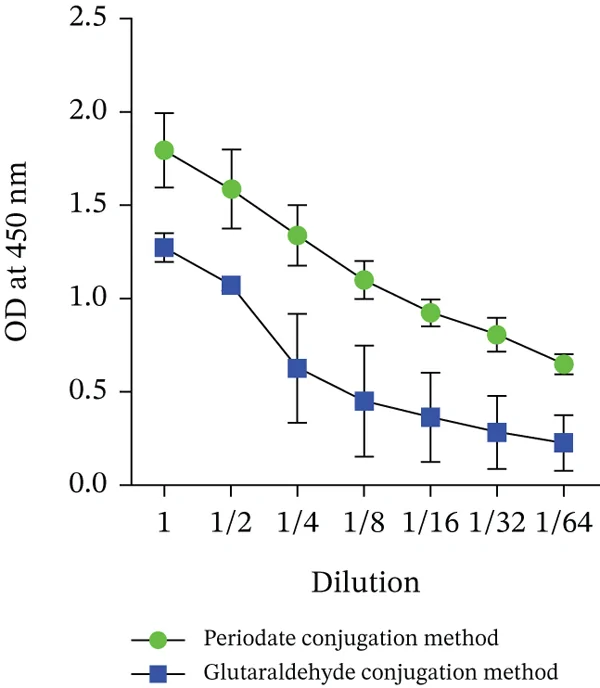
Comparative immunoreactivity of the periodate‐oxidised and the 12.5% glutaraldehyde IgY–HRP conjugates by direct ELISA. Symbols are the mean of the values obtained for each conjugate at each dilution; for the glutaraldehyde conjugate, error bars are the SD of two replicate wells. Wilcoxon matched‐pairs signed‐rank test across the seven dilutions, *p* = 0.0156.

In contrast, although the 12.5% glutaraldehyde conjugate performed better than the 25% version (as shown in the previous figure), it is still less effective than the periodate conjugate under these conditions. Statistical analyses were conducted to compare the performance of the glutaraldehyde conjugates at 12.5% with the periodate conjugation method. The Wilcoxon matched‐pairs signed‐rank test between the 12.5% glutaraldehyde conjugate and the periodate conjugate yielded a *p* − value of 0.0156, indicating a statistically significant difference (*p* < 0.05).

The results demonstrate that the three detection antibody systems (commercial biotinylated + streptavidin − HRP, glutaraldehyde‐conjugated IgY–HRP and periodate‐conjugated IgY–HRP) produce significantly different OD signals when tested on the same positive and negative samples (Figure 8). For positive samples, repeated measures ANOVA revealed a highly statistically significant difference among the three detection antibodies (*p* < 0.0001). Similarly, negative samples showed a highly significant difference between detection methods (*p* < 0.0001). Pairwise comparison with Bonferroni correction showed that on the positive sera, each system differed from the other two: the commercial system gave the highest mean signal, exceeding the glutaraldehyde conjugate by 0.167 OD units and the periodate conjugate by 0.082 OD units, and the periodate conjugate exceeded the glutaraldehyde conjugate by 0.085 OD units (all corrected *p* < 0.001). On the negative sera, the absolute differences were an order of magnitude smaller, at most 0.018 OD units; only the contrast between the two in‐house conjugates remained significant after correction (corrected *p* < 0.001), neither differing significantly from the commercial system (corrected *p* = 0.093 and *p* = 0.138). The background of both in‐house conjugates, therefore, remained at the level of the commercial reagent.

**Figure 8:**
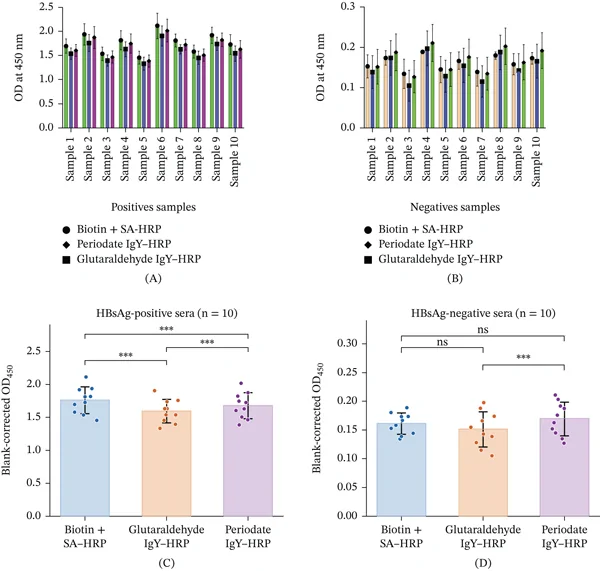
Diagnostic performance of the commercial biotinylated detection antibody and in‐house IgY–HRP conjugates in the adapted sandwich ELISA. Blank‐corrected OD450 values were compared for the commercial biotinylated detection antibody and IgY–HRP conjugates prepared by glutaraldehyde or periodate methods using (A) 10 HBsAg‐positive and (B) 10 HBsAg‐negative sera. Individual points/bars represent the mean of duplicate wells for each serum, assessed in two independent runs; group data are presented as mean ± SD. Statistical comparisons shown in (C) for positives samples and in (D) for negatives samples were performed using repeated‐measures ANOVA followed by paired post hoc tests with Bonferroni correction ( ^∗∗∗^, *p* < 0.001; ns, not significant).

ROC analysis against the Fortress Diagnostics reference classification confirmed that all three detection systems completely separated the 10 HBsAg‐positive from the 10 HBsAg‐negative sera, with no overlap in blank‐corrected OD450 values (Figure 9). Table 1 shown that mean signals for positive versus negative sera were 1.76 ± 0.202 versus 0.161 ± 0.018 (13.9‐fold) for the commercial biotin/streptavidin system, 1.593 ± 0.176 versus 0.151 ± 0.031 (15.8‐fold) for the glutaraldehyde conjugate and 1.678 ± 0.197 versus 0.169 ± 0.029 (14.6‐fold) for the periodate conjugate; differences were highly significant for all systems (Mann–Whitney *U* test, *p* < 0.0001). The area under the curve was 1.000 for each, with a maximal Youden index (J = 1.000) across the entire separation window. Midpoint cut‐offs were 0.82, 0.77 and 0.80 OD at 450 nm, respectively, for commercial biotinylated + streptavidin‐HRP, glutaraldehyde‐conjugated IgY–HRP and periodate‐conjugated IgY‐HR, although the conventional mean + 3 SD thresholds were substantially lower (0.216, 0.243 and 0.256) yet yielded identical classification. At either threshold, all systems achieved 100% sensitivity (10/10), 100% specificity (10/10) and 100% overall accuracy (20/20); the weakest positive serum had an S/CO ratio of ~6.72 and the strongest negative ~0.87, indicating a wide safety margin. Signal‐to‐noise ratios were 10.9 (biotin/streptavidin), 10.5 (glutaraldehyde) and 9.9 (periodate). However, with only 10 sera per group, the 95% Wilson CI on sensitivity and specificity is 72.2%–100%, and the cut‐offs are internal to this panel. These results demonstrate that the in‐house conjugates discriminate HBsAg status as effectively as the commercial reagent in this small set, but prospective validation on a larger, diverse cohort is required before clinical diagnostic claims can be made.

**Figure 9:**
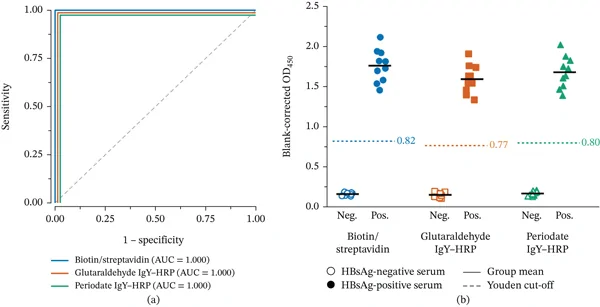
Diagnostic accuracy of the adapted sandwich ELISA with the commercial biotinylated detection system and with the two in‐house IgY–HRP conjugates, on 10 HBsAg‐positive and 10 HBsAg‐negative sera. (a) ROC curves against the Fortress Diagnostics reference classification: the three curves are identical, with an AUC of 1.000 each and are offset slightly from one another for visibility. (b) Blank‐corrected OD450 of the individual sera obtained with each detection system; the dashed lines mark the Youden‐derived cut‐offs.

**Table 1:** Diagnostic performance of the adapted sandwich ELISA with the commercial detection system and with the two in‐house IgY–HRP conjugates, on 10 HBsAg‐positive and 10 HBsAg‐negative sera classified by the Fortress Diagnostics reference ELISA. Cut‐offs are the values maximising the Youden index; the corresponding mean + 3 SD cut‐offs (0.216, 0.243 and 0.256) classify the panel identically. Confidence intervals are Wilson score intervals.

Detection system	Biotinylated Ab + streptavidin–HRP (kit reference)	IgY–HRP (glutaraldehyde)	IgY–HRP (periodate)
HBsAg‐positive sera, mean OD450 ± SD (*n* = 10)	1.760 ± 0.202	1.593 ± 0.176	1.678 ± 0.197
HBsAg‐negative sera, mean OD450 ± SD (*n* = 10)	0.161 ± 0.018	0.151 ± 0.031	0.169 ± 0.029
Positive/negative signal ratio	10.9	10.5	9.9
Highest negative OD450	0.189	0.198	0.211
Lowest positive OD450	1.454	1.333	1.386
Area under the ROC curve	1.000	1.000	1.000
Cut‐off, Youden index (OD450)	0.822^a^	0.765^a^	0.798^a^
Cut‐off, mean negative + 3 SD (OD450)	0.216	0.243	0.256
Sensitivity, % (95% CI)	100 (72.2–100)	100 (72.2–100)	100 (72.2–100)
Specificity, % (95% CI)	100 (72.2–100)	100 (72.2–100)	100 (72.2–100)
Accuracy, % (95% CI)	100 (72.2–100)	100 (72.2–100)	100 (72.2–100)
S/CO, weakest positive serum^b^	6.72	5.49	5.40
S/CO, strongest negative serum^b^	0.87	0.81	0.82

*Note:* Sensitivity, specificity and accuracy are identical under both cut‐off definitions (20/20 sera correctly classified).

^a^Separation between groups was complete, so every threshold between the highest negative and the lowest positive OD450 is tied at Youden J = 1; the value given is the midpoint of that interval.

^b^S/CO calculated against the mean negative + 3 SD cut‐off. Against the Youden cut‐off, the corresponding values are 6.72, 5.49 and 5.40 (weakest positive) and 0.87, 0.81 and 0.82 (strongest negative), respectively, for commercial biotinylated + streptavidin‐HRP, glutaraldehyde‐conjugated IgY–HRP and periodate‐conjugated IgY‐HR. The panel was too small and too cleanly separated to discriminate between them.

## 4. Discussion

Hepatitis B is the major health threat in Africa. The general objective of this study was to develop a robust, cost‐effective polyclonal anti‐HBsAg IgY‐based diagnostic platform for HBsAg detection, tailored for high‐prevalence, resource‐limited settings such as sub‐Saharan Africa.

The results of this study demonstrate the successful development of a polyclonal anti‐HBsAg IgY‐based diagnostic platform, from antigen‐specific immunisation through affinity purification and HRP bioconjugation to functional evaluation in a sandwich ELISA format. Structural integrity of the extracted IgY was confirmed by SDS‐PAGE, revealing the characteristic ~180 kDa band under non‐reducing conditions (the intact molecule) and the expected dissociation into 72 kDa heavy chains and 26 kDa light chains under reducing conditions. These profiles align precisely with established avian IgY biochemistry, where the slightly larger molecular weight and distinct glycosylation pattern compared with mammalian IgG (≈150 kDa) confer enhanced stability and resistance to proteolysis. The absence of significant contaminants in purified fractions further validates the modified salting‐out extraction (adapted from [16]) and subsequent affinity chromatography steps [16–18].

Immunoreactivity assessments via indirect ELISA confirmed that the crude yolk‐derived IgY already exhibited strong, dose‐dependent antigen recognition. This finding is consistent with prior observations that multivalent peptide immunisation enhances polyclonal avidity [10, 19]. Affinity purification using CNBr‐activated Sepharose coupled to the consensus peptides markedly enriched antigen‐specific IgY in the eluate fractions, as evidenced by high‐purity ~180 kDa bands on non‐reducing SDS‐PAGE and correspondingly elevated specific immunoreactivity (Figure 5). Although flow‐through fractions contained higher total protein (Bradford assay), they displayed negligible antigen‐specific signals, underscoring the necessity and efficacy of ligand‐specific affinity steps for removing non‐specific yolk proteins. This approach mirrors successful peptide‐based affinity purification strategies reported for other pathogen‐specific IgY preparations and highlights the value of consensus‐sequence antigens from the immunodominant “a” determinant (positions 99–169 of HBsAg) in mitigating immune‐escape variants, as previously emphasised in mutational analyses [10].

Bioconjugation of purified IgY to HRP produced functional enzyme‐antibody conjugates using both homobifunctional (glutaraldehyde) and heterobifunctional (NaIO₄ oxidation) chemistries. Direct ELISA titration curves demonstrated that the conjugate prepared with the 12.5% glutaraldehyde stock (0.124% final) outperformed the one prepared with the 25% stock (0.248% final) (Wilcoxon matched‐pairs signed‐rank test, *p* = 0.0312). The periodate‐oxidised conjugate consistently showed superior sensitivity and signal intensity across dilutions, with nearly twofold higher OD at starting concentrations compared with glutaraldehyde conjugates (Wilcoxon test, *p* = 0.0156).

Glutaraldehyde is a homobifunctional cross‐linker that primarily reacts with primary amine groups (mainly *ε*‐amino groups of lysine residues) on both the antibody and HRP. It forms covalent imine linkages, which can involve aldol condensation or Michael‐type addition mechanisms, resulting in both intermolecular and intramolecular crosslinks. At higher concentrations (e.g., 25% in this study), glutaraldehyde exhibits a strong tendency towards unregulated polymerisation during the crosslinking phase. This leads to the formation of large molecular complexes, a substantial proportion of which are insoluble polymers. Consequently, this reduces yield, causes loss of enzymatic activity (which can drop as low as 10% in one‐step methods), and promotes denaturation or masking of antibody paratopes and HRP active sites. Even when using a two‐step protocol, considerably high‐molecular‐weight aggregates can form that may precipitate from solution [15]. These effects explain the lower performance of the 25% glutaraldehyde conjugate compared with the 12.5% variant, which benefits from reduced over‐crosslinking, better preservation of binding sites and improved molar incorporation ratios. Different glutaraldehyde concentrations and treatment durations also produce varying degrees of antibody crosslinking, which can reduce epitope accessibility and binding affinity [20]. Overall, the random amine‐targeted crosslinking of glutaraldehyde often results in uncontrolled polymerisation, high non‐specific aggregation and lower retention of biological activity.

In contrast, the NaIO₄ method is directional and site‐specific. The reagent selectively oxidises the vicinal diols in the carbohydrate moieties of HRP (which constitute ~8%–10% of the enzyme by weight) to generate multiple reactive aldehyde groups, while largely preserving the enzyme′s heme catalytic site. These aldehydes then react with primary amine groups (mainly lysine residues) on the IgY antibody under mildly alkaline conditions (typically pH 9.0–9.6 in sodium bicarbonate buffer), forming reversible Schiff base (imine) linkages. Excess aldehydes and periodate are quenched (e.g., with glycine, ethanolamine or glycerol). This process is often described as reductive amination, though additional borohydride reduction is not always necessary in optimised protocols [21].

This approach provides more coupling sites and higher labelling efficiency than glutaraldehyde amine‐targeted crosslinking. As a result, it achieves greater retention of enzymatic activity, reduced non‐specific aggregation and improved signal‐to‐noise ratios in ELISA applications. These advantages make the periodate method particularly suitable for diagnostic reagents requiring maximal sensitivity [12, 13].

When integrated as detection reagents in an adapted commercial sandwich ELISA (Figure 8), both in‐house IgY–HRP conjugates produced statistically distinct OD profiles from the manufacturer′s biotinylated/streptavidin‐HRP system on the same positive and negative clinical sera (repeated‐measures ANOVA, *p* < 0.0001 for both sample types). Positive samples generated robust signals with the periodate conjugate, whereas negative samples remained low, confirming retained specificity. These performance differences underscore that conjugation chemistry directly modulates assay sensitivity and that optimised IgY–HRP reagents can rival or surpass commercial mammalian antibody systems in discriminatory power. The results confirm earlier demonstrations of anti‐HBsAg IgY utility in ELISA formats, where HRP‐labelled chicken antibodies successfully detect HBsAg without mammalian serum interference [3, 6]. The higher reactivity observed with commercial biotinylated + streptavidin‐HRP than with glutaraldehyde‐conjugated IgY–HRP and periodate‐conjugated IgY–HRP can be explained by several factors: Assay signal generally increases when the number or accessibility of binding interactions is enhanced, particularly when multiple epitopes can be engaged simultaneously or repeatedly [22]. Multivalent or multiepitope configurations boost avidity by allowing both antibody binding sites to engage and often outperform single‐epitope formats, as shown with commercial biotinylated + streptavidin‐HRP; epitope‐resolved digital profiling has shown improved sensitivity over single‐epitope readouts by maintaining distinct epitope‐specific signals, and tandem multiepitope display has yielded roughly twofold higher binding capacity than linear or branched alternatives, whereas sandwich binding for small targets remains uncommon and requires precise spacing and recognition of non‐overlapping epitopes [23, 24]. In parallel, conventional nonspecific conjugation tends to generate heterogeneous products and can modify regions proximal to antibody binding domains, impairing performance; random lysine or cysteine coupling produces mixed species with reduced reproducibility and can diminish affinity and specificity when CDRs or paratopes are affected [25, 26]. By contrast, site‐specific and enzyme‐directed conjugation strategies were developed to minimise such functional losses, yielding more homogeneous conjugates with preserved activity and engineered or chemoenzymatic approaches have been reported to leave affinity largely unchanged or to retain target‐cell binding after modification [25, 27].

The present work builds upon and advances prior IgY‐based HBV diagnostics by incorporating consensus peptides from the MHR (selected for high immunogenicity and escape‐mutation resistance). It also rigorously compares two widely used conjugation chemistries. Unlike mammalian IgG, IgY offers substantial practical advantages for resource‐limited settings: non‐invasive harvesting from eggs (yielding gramme quantities per hen annually), negligible cross‐reactivity with human complement, rheumatoid factor, or Fc receptors and reduced background signals in human sera. These attributes minimise false positives in endemic regions such as sub‐Saharan Africa, where HBV prevalence is high and diagnostic infrastructure is constrained. Cost‐effectiveness is further enhanced using low‐volume Freund′s adjuvant immunisation and scalable yolk extraction, consistent with the economic rationale for IgY platforms reported across viral diagnostics [5, 28, 29].

Several limitations should be emphasised. First, the clinical panel comprised only 20 sera (10 HBsAg‐positive and 10 HBsAg‐negative). Although the assay classified every sample correctly and the ROC analysis gave an AUC of 1.000 for all three detection systems, a panel of this size supports a 95% CI for sensitivity and specificity of only 72.2%–100%, and the cut‐off reported here was derived from, and is therefore internal to, the same panel. Validation on a larger, independent and consecutively recruited cohort ideally including a WHO international standard, seroconversion panels, low‐titre specimens and sera carrying documented “a”‐determinant escape mutations is required before any claim of clinical accuracy can be made and is the immediate priority for follow‐up work. Second, the panel contained no specimens with potentially interfering conditions (rheumatoid factor, hyperbilirubinaemia, haemolysis, HIV or HCV coinfection), so the specificity advantage expected of avian IgY in human matrices remains to be demonstrated directly. Third, the molar labelling degree of the conjugates (HRP molecules per IgY) was not determined; the conjugates were compared functionally rather than stoichiometrically, and spectrophotometric and size‐exclusion characterisation of the labelling degree would strengthen the mechanistic interpretation of the difference between the two chemistries. Fourth, long‐term stability of the conjugates was not assessed. Potential batch‐to‐batch variability in polyclonal IgY, although inherent, can be mitigated by pooling from multiple hens or transitioning to monoclonal IgY lines. Nonetheless, the statistically robust discrimination of positive versus negative sera, combined with the superior performance of the periodate conjugate, supports the feasibility of this IgY platform as a low‐cost, locally producible alternative for HBV screening.

## 5. Conclusion

This study successfully established a complete pipeline for producing and optimising polyclonal anti‐HBsAg IgY antibodies for diagnostic applications. From consensus peptide design and hen immunisation through IgY extraction, affinity purification and HRP bioconjugation, the workflow yielded functional reagents capable of detecting HBsAg in human serum. Structural integrity was confirmed by SDS‐PAGE, specific immunoreactivity by indirect ELISA and superior performance by the NaIO₄ oxidation conjugation method in direct and sandwich ELISA formats.

Key insights emerged regarding both the platform and the chemistry. The use of a peptide mixture targeting the immunodominant “a” determinant in the MHR elicited strong polyclonal responses, whereas affinity purification effectively enriched antigen‐specific IgY, removing the bulk of non‐specific yolk proteins. In bioconjugation, the periodate method consistently outperformed glutaraldehyde (particularly at the optimised 12.5% concentration), delivering nearly twofold higher signal intensity and better preservation of both antibody binding and enzymatic activity. These results highlight the importance of controlled oxidation and reductive amination for achieving high‐labelling efficiency with minimal denaturation in avian antibody systems.

The significance of this work lies in its potential to provide a robust, low‐cost and locally producible diagnostic alternative for hepatitis B screening. IgY offers distinct advantages over mammalian antibodies, including high yield from eggs, negligible interference with human serum components and reduced background noise, particularly valuable in high‐prevalence, resource‐limited settings such as sub‐Saharan Africa. By demonstrating the statistically superior performance of in‐house IgY–HRP conjugates compared with commercial biotin‐streptavidin systems in clinical samples, this platform supports the development of affordable, high‐quality immunoassays that can help address diagnostic gaps in endemic regions.

Future efforts should focus on scaling production, conducting larger clinical validation studies against international reference panels and assessing the long‐term stability of the conjugates. Transitioning towards recombinant or monoclonal IgY formats could further reduce batch variability. Coupling affinity‐purified IgY to the signal‐amplification chemistries now being developed for nucleic‐acid biosensors is a further avenue for increasing analytical sensitivity without abandoning the cost and stability advantages of an antibody‐based format. Overall, this IgY‐based approach represents a promising step towards sustainable, locally driven solutions for HBV diagnosis, with broader applicability to other infectious disease targets.

## Author Contributions

F.T.T.: conceptualisation, data curation, formal analysis, investigation, methodology, resources, writing original draft. H.P.N‐N.: conceptualisation, data curation, investigation, methodology, resources, software, supervision, validation, writing original draft, writing review and editing. Y.F.: formal analysis, supervision, resources, validation, writing review and editing. F.T.M.: data curation, formal analysis, investigation, software, supervision, visualisation, writing original draft. A.F.K.: formal analysis, supervision, resources, validation, writing review and editing. S.N.F.: data curation, formal analysis, supervision, writing review and editing. F.N.N.: formal analysis, resources, supervision, visualisation, writing review and editing. P.F.M.: conceptualisation, resources, supervision, visualisation, writing review and editing. F.T.T. and H.P.N‐N. contributed equally to this work.

## Funding

No funding was received for this manuscript.

## Disclosure

All authors have read and agreed to the published version of the manuscript.

## Ethics Statement

This study was approved by the Ethics and Quality Assurance Committee (Comité d′Ethique et d′Assurance Qualité) of l′Université des Montagnes (Approval No.: 2022/192/UdM/PR/CEAQ) and adheres to the ethical standards for animal welfare. The study was conducted in accordance with the recommendations of the University of Yaoundé 1 Animal Ethics Committee.

## Consent

The authors have nothing to report.

## Conflicts of Interest

The authors declare no conflicts of interest.

## Data Availability

The data that support the findings of this study are available from the corresponding author upon reasonable request.
